# Metabolic alterations in meningioma reflect the clinical course

**DOI:** 10.1186/s12885-021-07887-5

**Published:** 2021-03-01

**Authors:** Waseem Masalha, Karam Daka, Jakob Woerner, Nils Pompe, Stefan Weber, Daniel Delev, Marie T. Krüger, Oliver Schnell, Jürgen Beck, Dieter Henrik Heiland, Juergen Grauvogel

**Affiliations:** 1 Department of Neurosurgery, University Medical Center Freiburg, Breisacher Straße 64, 79106 Freiburg, Germany; 2 Faculty of Medicine, University of Freiburg, Freiburg im Breisgau, Germany; 3 Institute of Physical Chemistry, Faculty of Chemistry and Pharmacy, University of Freiburg, Freiburg im Breisgau, Germany; 4 Department of Neurosurgery, RWTH University, Aachen, Germany; 5 Department of Neurosurgery, Cantonal Hospital St.Gallen, st. gallen, Switzerland

**Keywords:** Tumour metabolism, Meningioma

## Abstract

**Background:**

Meningiomas are common brain tumours that are usually defined by benign clinical course. However, some meningiomas undergo a malignant transformation and recur within a short time period regardless of their World Health Organization (WHO) grade. The current study aimed to identify potential markers that can discriminate between benign and malignant meningioma courses.

**Methods:**

We profiled the metabolites from 43 patients with low- and high-grade meningiomas. Tumour specimens were analyzed by nuclear magnetic resonance analysis; 270 metabolites were identified and clustered with the AutoPipe algorithm.

**Results:**

We observed two distinct clusters marked by alterations in glycine/serine and choline/tryptophan metabolism. Glycine/serine cluster showed significantly lower WHO grades and proliferation rates. Also progression-free survival was significantly longer in the glycine/serine cluster.

**Conclusion:**

Our findings suggest that alterations in glycine/serine metabolism are associated with lower proliferation and more recurrent tumours. Altered choline/tryptophan metabolism was associated with increases proliferation, and recurrence. Our results suggest that tumour malignancy can be reflected by metabolic alterations, which may support histological classifications to predict the clinical outcome of patients with meningiomas.

**Supplementary Information:**

The online version contains supplementary material available at 10.1186/s12885-021-07887-5.

## Background

Meningiomas are common brain tumours in adults; they account for 13–26% of all intracranial tumours [1]. The 2016 World Health Organization (WHO) classification divided meningiomas into three histological grades (I to III) and described 16 histopathological subtypes [2]. These WHO grades have presented a significant correlation with clinical outcomes in several recent studies. The majority of meningiomas are benign tumours (90%) that are mainly treated by surgery, followed by an innocent clinical course [1]. Atypical meningiomas (WHO grade II) reveal a worse clinical outcome due to higher recurrence rates of up to 30–40% [2]. In addition, a small subset of meningiomas (1–2.8%) is classified as anaplastic; they show a particularly aggressive clinical course and a recurrence rate of nearly 100% [1].

A few benign meningiomas (WHO grade I) can transform into an aggressive growth pattern and recur after a short period of time. To address the variety of clinical outcomes within the WHO grade I group, multiple research groups have investigated the genetic landscape of meningiomas and have shown region-specific genomic alterations [3]. The authors have identified five genetic subgroups based on transcriptional similarity and associated mutational patterns. Meningiomas most frequently contained an *NF2, SMARCB1* (Group1)*, TRAF7/KLF4* (Group2), and *PI3K* (Group3) mutation, which drive WNT pathway activation. In another group, there was Hedgehog pathway activation detected by transcriptional profiles and mainly based on mutations in genes such as *SMO* [4]*.* The last subgroup comprised meningiomas that showed a distinct mutation in the *POLR2A* gene followed by dysregulated RNA synthesis [3, 5]. others suggested that malignant meningiomas have different DNA methylation patterns than other types of meningiomas and that the differently methylated genes could serve as diagnostic biomarkers for malignant transformation [6]. The importance of TERT promoter mutations and their potential use as biomarkers to identify meningiomas at risk of malignant transformation was also reported [7].

Besides genomic alterations, tumour metabolism has recently been described as a hallmark driver of malignancy and tumour development. Different tumour entities, including brain and other solid tumours, have shown numerous metabolic alterations. A strong association between altered tumourmetabolism and chromosomal instability has been reported [8]. These metabolic alterations were detected independently of the WHO grade [9, 10]. Serna et al. [11] showed that metabolic aggressiveness is driven by alterations in the expression of *IGF1R* (insulin-like growth factor 1 receptor), which is involved in the regulation of glycolysis. Following the hypothesis of altered glycolysis in meningiomas, researchers have shown that different components of glycolysis are transformed, including phosphofructokinase (PFK) and lactate dehydrogenase (LDH), both of which were significantly increased in anaplastic meningioma compared with other histological subtypes. In addition, there were alterations in tryptophan metabolism, a phenomenon that forced the immune-escape mechanism to increase kynurenine pathway activity [12, 13]. Today, increased opportunities in bioinformatics and metabolic profiling have allowed detecting associations between metabolic networks and clinical parameters to predict metabolic patterns and associated clinical outcomes. Our aim was to identify benign and potentially malignantly transformed meningiomas within the defined WHO grade I by metabolic profiling and computational analysis.

## Methods

### Contact for reagent and resource sharing

Further information and requests for resources, raw data, and reagents should be directed to and will be fulfilled by DH Heiland (dieter.henrik.heiland@uniklinik-freiburg.de). A full table of all materials is given in the supplementary information.

### Ethical approval

For this study, we included 43 patients who underwent surgery at the Department of Neurosurgery of the University Medical Center Freiburg. The local ethics committee of the University of Freiburg approved data evaluation, imaging procedures, and the experimental design (protocols 100,020/09 and 5565/15). The methods were carried out in accordance with the approved guidelines. Written informed consent was obtained from each patient. The studies were approved by an institutional review board.

### Imaging, tissue collection, and histology

Tumour tissue was sampled from the meningioma core, snap-frozen in liquid nitrogen immediately after resection, and processed for further metabolic analysis. Representative tissue from all samples were fixed using 4% phosphate-buffered formaldehyde and embedded in paraffin following standard procedures. Hemotoxylin and eosin (H&E) staining was performed on 4 μm paraffin sections using standard protocols. This staining confirmed the correct sampling.

### Metabolite extraction and hydrogen nuclear magnetic resonance (^1^H-NMR) analysis

Metabolites were extracted with 400 μL ice-cold 80% methanol and 400 μL ice-cold water, homogenized with a tissue grinder (VWR, Radnor, PA, USA), sonicated at 1 °C, then centrifuged at 15,000 *g* for 20 min to remove protein. Extracts were dried by lyophilization and resuspended in 650 μL deuterated water as described by Beckonert et al [14] Six-hundred microliters of the suspension was transferred to NMR tubes for the subsequent NMR procedure. ^1^H-NMR spectra were collected at the Institute of Physical Chemistry of the University of Freiburg with a Bruker Avance III HDX 600-MHz NMR spectrometer (Bruker, Rheinstetten, Germany), equipped with a PABBO BB/19F-1H/D Z-GRD probe head. Each individual spectrum was recorded with two dummy scans and 32 scans with 64 k points in the time domain. The sweep width was set to 16.02 ppm with an offset of 4.691 ppm. This resulted in an acquisition time of 3.4 s for each scan and a dwell time of 52 microseconds. The relaxation delay was set to 2 s for acquisition, and the water signal was suppressed by an excitation sculpting scheme [15].

### Postprocessing of metabolic data

To adjust the spectra from multiple batches, spectra were manually aligned by setting the peak of l-lactic acid at 1.310 ppm. All acquisition and processing of the spectra were performed with TopSpin 3.2 patch level 6. A detailed description of the methods was given in a recently published study by Heiland et al [16] All spectra were analyzed with the software package “batman,” an R-software-based tool for metabolite detection in complex spectra [17]. This tool fits a predefined list of metabolites by a Bayesian approach. Hao et al. [17] provided a detailed description of the batman algorithm. Normalization of the spectra was performed by the pseudo-counted quantile (pQ) normalization algorithm integrated in the KODAMA package. Further processing of metabolic data is described in the subsequent subsections.

### Cluster analysis

Normalized metabolic data were processed with AutoPipe (https://github.com-/heilandd/AutoPipe), a software package for automated unsupervised clustering. First, the number of subgroups was computed by the Partitioning Around Medoids (PAM) algorithm (Cluster number *k* = 2–12). To identify the optimal number of clusters, we calculated the mean silhouette width of each cluster composition. Next, to identify the core samples of each cluster, we removed samples with a negative silhouette width from further analysis. We then used either the PAMR algorithm [18], a machine-learning-based method, or a generalized linear model [19] to identify characteristic up- or downregulated metabolites of each subgroup.

### Weighted correlation network analysis (WCNA)

WCNA is a robust tool for integrative network analysis and has been used in recent studies [20–22]. It is based on a scaled-topology-free-based network approach and uses the topological overlapping measurement to identify corresponding modules. These modules were analyzed by their eigengene correlation to each metabolite. The correlation of the intramodular connectivity (kME) and metabolites was used as input for a “Cluster of Clusters Analysis.” This analysis integrates expression modules and metabolites, which presents equal correlation values (kME and metabolite intensity values). A detailed description of WCNA is given in a previous publication [23].

### Functional analysis

Metabolic data was processed by pathviewer, an R package that includes Kyoto Encyclopedia of Genes and Genomes (KEGG) pathway maps [24]. Expression data (as described above) and normalized, log2-transformed, and median-centered metabolic data were integrated in the pathviewer algorithm. Enrichment analysis of metabolic data was performed with the DOSE package and the web-based tool MetaboAnalyst 3.0 (www.metaboanalyst.ca).

### Survival analysis

Progression-free survival analysis was conducted using Kaplan-Meier analysis, with the betweed-cluster differences analyzed using a log-rank test.

## Results

### Meningiomas revealed two distinct metabolic clusters

We started our investigation by purifying metabolites from 43 meningiomas localized at different anatomical regions, including the convexity in 12 cases (27.9%), the falcine site in 6 cases (13.9%), the sphenoid ridge in 6 cases (13.9%), the frontobasal region in 9 cases (20.9%), the petroclival region in 2 cases (4.6%), the spinal site in 3 cases (6.9%), and 5 cases at other regions. The histopathological analysis showed 28 patients with WHO grade I meningiomas (65.1%), 12 with WHO grade II meningiomas (27.9%), and 3 patients with WHO grade III meningiomas (6.9%). Two patients with a WHO grade II meningioma and 3 patients with a WHO grade III meningioma showed a tumour recurrence within 1 year. Twenty-four (55.9%) patients had a proliferation index (MIB-1) below 5% and 19 (44.1%) patients had a proliferation index (MIB-1) above 5%. A gross total resection (Simpson grade 1 + 2) was achieved in 37 patients and a subtotal resection (Simpson grade 3 + 4) was achieved in 6 patients. A detailed overview of all parameters can be found in Table 1.

**Table 1:** Patients data

Parameter	Cluster 1 *N* = 15	Cluster 2 *N* = 28	Significance level (p)
Sex (N,%)			
Male (*N* = 10)	2 (14.2%)	8 (28.5%)	0.45****
Female (*N* = 33)	13 (86.6%)	20 (71.4%)	
Age (mean, SD)	62.3 ± 13.1	64.4 ± 12.8	0.62*
Size (cm^3^) (mean, SD)	48.3 ± 51.8	88.6 ± 103.3	0.09*
Peritumoral edema (N, %)			
high	4(26.6%)	18(64.3%)	0.02***
low	11(73.4%)	10(35.7%)	
Location (N, %)			
falx	3(20%)	3(10.7%)	0.64****
convexity	2(13.3%)	10(35.7%)	0.16****
frontobasal	4(26.6%)	5(17.8%)	0.69****
sphenoid wing	2(13.3%)	4(14.2%)	1***
petroclival	1(6.6%)	1(3.5%)	1****
spinal	2(13.3%)	1(3.5%)	0.11****
others	1(6.6%)	4(14.2%)	0.64****
Simpson grade (N, %)			
Grade 1	5(33.3%)	7(25%)	0.74****
Grade 2	9(60%)	16(57.1%)	1***
Grade 3	0(0%)	3(10.7%)	0.54****
Grade 4	1(6.6%)	2(7.1%)	1****
Preoperative KPS (median, 95% CI)	80% CI(70–80%)	70% CI(%60–80%)	0.01**
Postoperative KPS (median, 95% CI)	90% CI(80–90%)	70% CI(50–90%)	0.007**
Proliferation index (N, %)			
MIB1 < 5%	15(100%)	9(32.1%)	0.01****
MIB1 > 5%	0(0%)	19(67.9%)	
Pathology (N, %)			
WHO grade I	15(100%)	13(46.4%)	0.001****
WHO grade II + III	0(0%)	15(53.6%)	

*SD* standard deviation

*CI* confidence interval

*KPS* Karnofsky score

* T-test

**Wilcoxon test

***Chi-squared test

****Fisher’s exact test

**Fig. 1:**
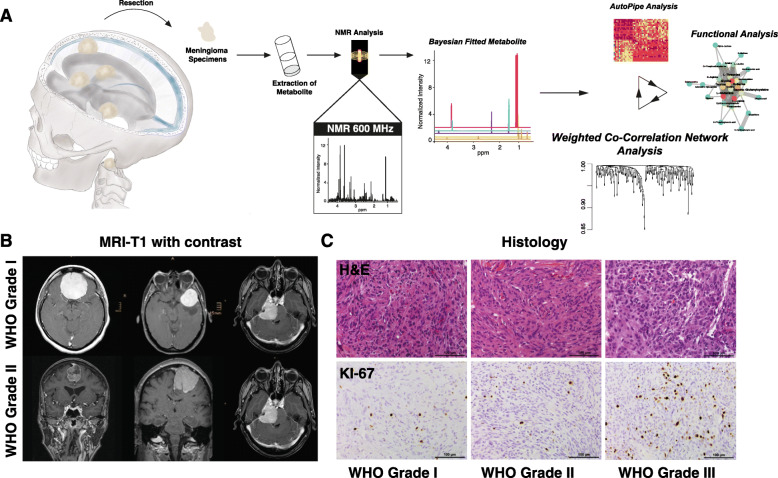
**a** Workflow of metabolite extraction, identification of metabolite and bioinformatical analysis. The extracted specimens are analyzed by NMR analysis. Metabolite are fitted and further analyzed by unsupervised clustering and network analysis. **b** T1-weighted MRI images with contrast are illustrated. **c** Representative H&E stainings of WHO grade I-III (upper panel) and KI-67 immunostainings (bottom panel) of WHO grade I-III

**Fig. 2:**
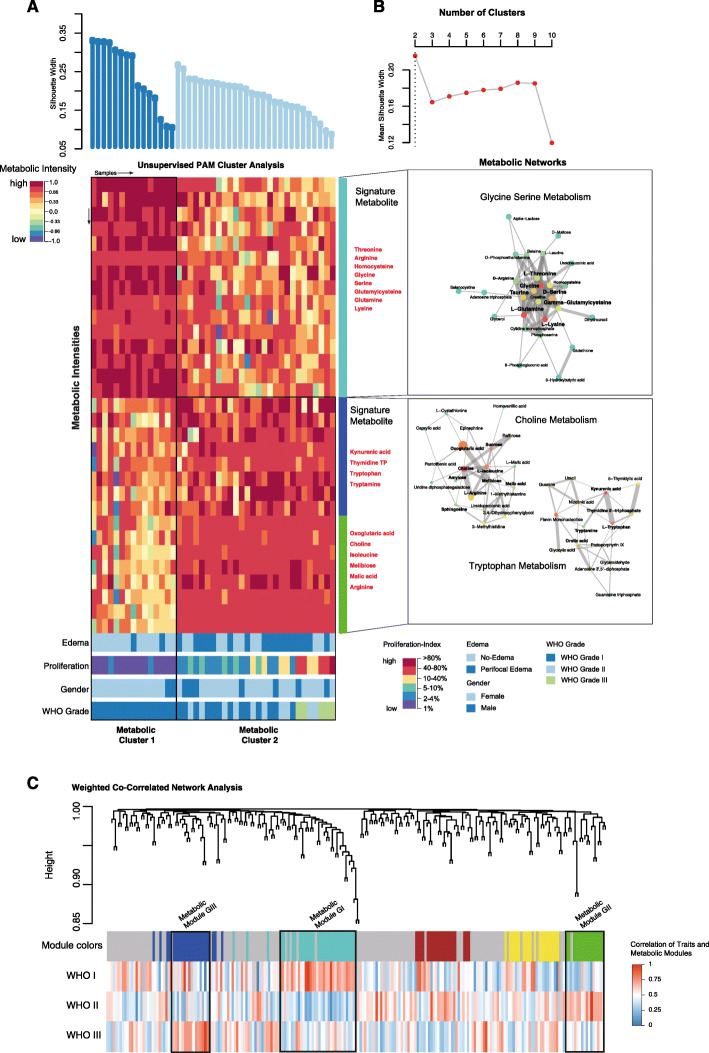
**a** In the upper plot, the silhouette widths of both clusters are shown ordered by different cluster subgroups. In the middle panel, a heatmap illustrate the metabolic intensity of the top ranked metabolites. At the bottom panel, the information on clinical properties is available. These are color-coded and explained in the side panel. **b** In the upper panel, the plot illustrates the optimal number of clusters. The optimal number of clusters was achieved by PAM clustering from 2 to 10 number of clusters by calculating the mean silhouette widths. In the bottom panel, a network analysis of the metabolic pathways highly enriched in cluster 1 and 2 respectively is given. **c** A weighted correlation network analysis (WGNC) was performed to explore the associated clinical features and molecular/metabolic, further detailed interpretation of all identified modules is given in the supplementary data

**Fig. 3:**
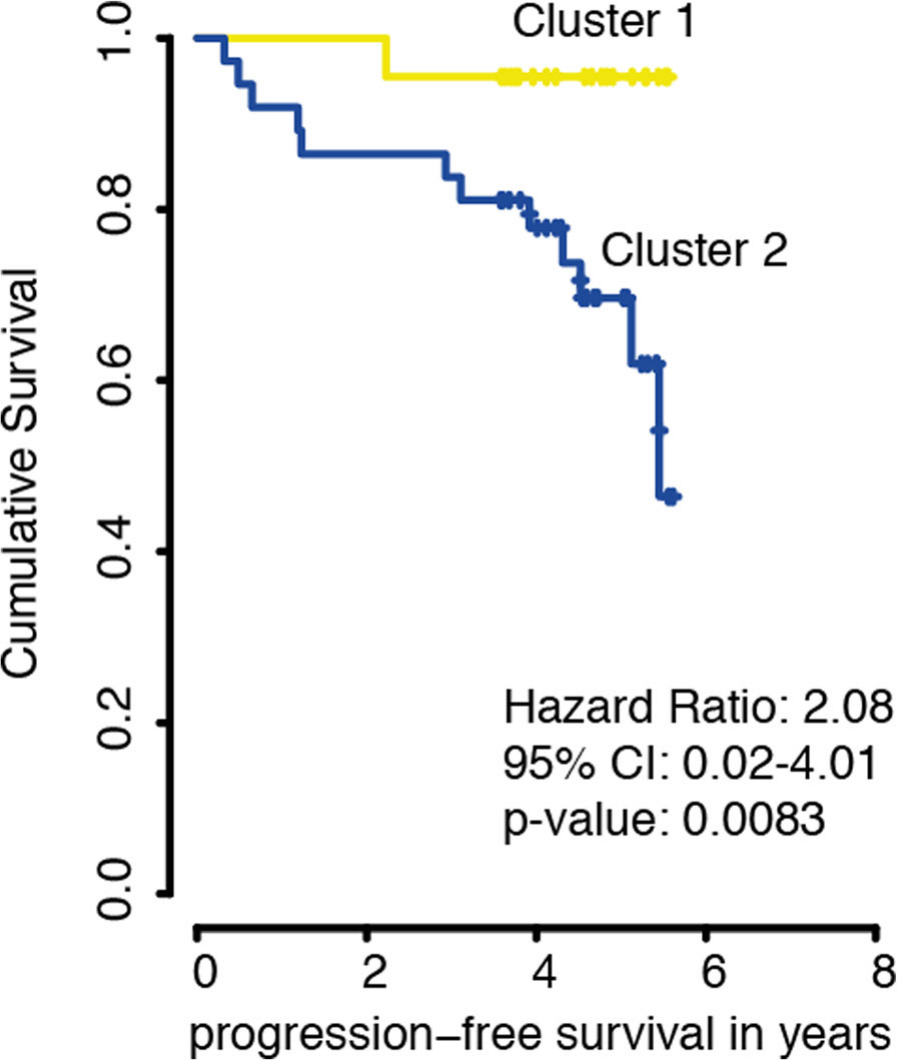
Kaplan-Meier Kurve for Prgression-free survival based on different Clusters (Cluster 1 in yellow vs Cluster 2 in blue)

Metabolites were analyzed using NMR and processed by a comprehensive computational analysis (Fig. 1). Representative MRI and histological staining’s are illustrated in Fig. 1b-c. We first conducted an unsupervised cluster analysis of the top 100 most variable metabolites by PAM clustering. The optimal number of clusters was defined by maximal mean silhouette widths (Fig. 2a, b). The analysis revealed two distinct clusters. In the first cluster, named Metabolic Cluster I, we found upregulated glycine/serine metabolism with the major signature metabolites glycine, serine, and arginine. Patients in this cluster were exclusively histological grade I and showed a significantly lower rate of edema (*p* < 0.05) and a low proliferation rate (mean 1.2, interquartile range [IQR] 0.3, *p* < 0.05) compared to cluster II. The second cluster (Metabolic Cluster II) was easily separable into two subclusters by PAM clustering. One subcluster contained patients with a medium proliferation rate of 2.1 (IQR 0.7) and increased edema compared with Cluster I (*p* < 0.05; Fig. 2a, b). The pathway that separated the clusters was choline metabolism. For the second subcluster, we found increased tryptophan metabolism and also a strong activation of the choline pathway. Furthermore, the proliferation rate was massively increased (mean 11.7, IQR 4.3, *p* < 0.05), and there was edema in most of the patients compared to patients in cluster I. We found a high incidence of meningioma WHO grade II (*n* = 12) and III (*n* = 3) but also meningioma WHO grade I (*n* = 13) (Fig. 2a, b). Kaplan-Meier analysis showed a significant longer progression-free survival in favour of cluster I (*p* = 0.0083), suggesting that metabolic alterations may reflect the clinical course of the disease, Fig. 3.

### WCNA of metabolic profiles in Meningiomas

We aimed to characterize the metabolic pathways in detail and performed a WCNA of metabolic profiles. There were five metabolic modules (Fig. 2c). Three modules showed a strong association with the WHO grade based on a logistic model that included WHO grade and module eigengene. From that model, we considered module GI (associated with WHO grade I, *R* = 0.25, *p*_adj_ = 0.05), module GII (associated with WHO grade II, *R* = 0.26, *p*_adj_ = 0.05), and module GIII (associated with WHO grade III, *R* = 0.28, *p*_adj_ = 0.01) for further analysis. The other modules failed to achieve a significant correlation with the WHO grade. First, we analyzed the correlation between the GI module eigengene and clinical features (Figure S1A). We found a significant negative correlation between module GI and perifocal edema (*R* = − 0.3, *p*_adj_ = 0.01) and the histologically defined atypical meningiomas (*R* = − 0.38, *p*_adj_ = 0.03), which confirmed our NMR findings. Meningiomas with a module GI signature were mostly observed in the convexity (*R* = − 0.32, *p*_*adj*_ = 0.04). The histological subtype of a “transitional meningioma” was highly significantly associated with the GI signature (*R* = − 0.41, *p*_*adj*_ = 0.001). Next, we examined the enrichment of metabolic pathways in each module. A metabolite set enrichment analysis (MSEA) showed a significant upregulation of the glycine/serine pathway (Enrichment Score = 1.75, *p* = 0.0067) and glutamate metabolism (Enrichment Score = 1.52, *p* = 0.047) (Figure S1B). We computed a network based on the metabolites of module GI. Connections within the network were based on intramodular connectivity. From that network, we identified the amino acids serine and glycine and the metabolite creatine as key metabolic players in grade I meningiomas.

In the next step, we examined the correlation between the GII module eigengene and clinical features (Figure S2A). There was a significant correlation between module GII and perifocal edema (*R* = 0.25, *p*_*adj*_ = 0.05) and the histologically defined atypical meningioma (*R* = 0.41, *p*_*adj*_ = 0.02). There was no correlation between localization and the module eigengene. The metabolic network of module GII identified tryptophan and kynurenic acid as hub metabolites (Figure S2B). In line with the network findings, a metabolic pathway analysis uncovered upregulation of the tryptophan metabolism pathway (Enrichment Score = 1.83, *p* = 0.0093; Fig. 2c). To characterize module GIII, we examined the correlation between module eigengene and clinical features (Fig. S2D). No clinical feature was associated with this module, except the WHO grade (*R* = 0.28, *p*_*adj*_ = 0.01). In addition, there was no correlation between localization and module eigengene. We next computed a network of metabolites for module GIII. Choline, iso-leucine, and sphingosine were hub metabolites, (Figure S2E). In line with the network findings, a metabolic pathway analysis uncovered an upregulation of the choline metabolism pathway (Enrichment Score = 1.06, *p* = 0.0014).

## Discussion

Due to a favorable clinical course, most meningiomas are classified as benign. Nevertheless, 20% of patients within this subgroup present a tumour recurrence, even after gross total resection [1, 25]. These tumour subtypes are difficult to identify and challenging for future treatment. To better predict their clinical outcome, researchers have performed metabolic analysis by high resolution magic angle spinning (HR-MAS) and reported a correlation between metabolic and WHO graduation of meningiomas [9]; however, these findings were limited by a small number of observed metabolites. We aimed to investigate a global metabolic profiling to identify specific subgroups that are driven by unique metabolic alterations and associated with WHO grades. We performed ex vivo metabolic profiling by NMR spectroscopy of meningioma specimens, including all WHO grades (I, II, and III), followed by computational detection of metabolites and cluster analysis.

Our cluster analysis identified two major metabolic clusters, of which one cluster was divided into two subclusters of meningiomas based on the top 100 most variable metabolites. The first metabolic cluster was highlighted by serine and glycine metabolism, which provides a basis for synthesis of other amino acids, proteins, and lipids. Their metabolism could increase the antioxidative capacity and reduce cellular reactive oxygen species (ROS). In particular, the glycine cleavage system fuels mono-carbon metabolism within a complex cellular network based on folate compound reactions, which play a central role in cellular redox balance over modulation of the NADP^+^/NADPH-ratio [26]. This network is also an essential component of methylation reactions that may be relevant for the maintenance of the cellular epigenetic status [27]. In our study, serine and glycine metabolism was associated with benign meningiomas (WHO grade I) and a significantly low proliferation index (*p* = 0.01). This cluster was also accompanied by significantly lower edema based on magnetic resonance imaging (*p* = 0.02; Fig. 2, Table 1) compared with higher grades. The role of glycine in meningiomas has been controversially discussed. While some authors have reported a complete absence of glycine in aggressive meningioma [28], others have shown a significantly increased glycine/alanine ratio. Furthermore, a significantly increased ratio of glycine to glutamine/glutamate in rapidly recurrent meningiomas and an increased glycine value in benign meningioma has been observed [29, 30]. By contrast, Monleon and colleagues found increased glycine levels in atypical meningiomas due to hypothesized increased angiogenesis [9]. These discrepancies with regard to glycine levels suggest the existence of more complex subgroups within the pathological delineation of grade I meningiomas. In line with published data, we detected altered glycine/serine metabolism in a subgroup of benign meningiomas. Other clusters also contain grade I tumours, which results in inaccurate individual metabolic levels within grade I meningiomas.

The second metabolic cluster was defined by choline and tryptophan metabolism. Choline is an important component of the cell membrane. Higher choline concentrations are usually present in brain tumours compared with normal brain tissue. Thereby, choline acts as a brain tumour marker [31]. Elevated choline peaks can also be detected in regions with high tumour cell density, in which the tissue is not necessarily anaplastic [32]. The increased rate of proliferation, which we observed in tumours of the second cluster, consequently drives the choline metabolism due to increased demand of membrane components. Previous findings have suggested that choline levels are significantly increased in benign meningioma with an aggressive clinical course [9, 11]. Further, Mishra and colleagues reported high choline levels in malignant meningioma [33]. By contrast, others have reported a prominent choline level in WHO grade I meningioma. While they did not observe differences in choline concentrations within all WHO grades, they only included a small number of WHO grade II + III meningiomas in their study [29]. We further identified altered tryptophan metabolism; this amino acid is mainly metabolized by three pathways: the serotonin (5-hydroxytryptamine [5-HT]) pathway, the kynurenine (KYN) pathway, and the tryptamine pathway [34]. Researchers have reported that an overexpression of the tryptophan catabolizing enzymes indoleamine 2,3-dioxygenase 1/2 or tryptophan 2,3-dioxygenase are associated with tumour progression. These two enzymes convert tryptophan into kynurenine [35, 36], which is involved in the immune escape mechanism [37, 38]. This indicates that the tryptophan and the kynurenine pathways play an important role in tumour progression and immune response. Researchers have confirmed the increased tryptophan metabolism in high-grade meningiomas [12, 13]. In line with the reported findings, we confirmed the increased tryptophan metabolism in high-grade meningiomas (grade II and III) but also identified benign meningiomas with upregulated choline and tryptophan metabolism. These tumours were significantly associated with a shorter PFS in our study (*p* = 0.0083) (Fig. 3). This insight provides a possible rationale suggesting that some benign classified meningioma manifest a less favorable course than others.

Taken together our findings suggest an important role for tumour metabolism in meningiomas that only partially overlaps with respect to their histological grading. We addressed the questions as to how metabolic changes in meningiomas can provide a possible explanation for different clinical features within these histologically classified tumours. Our metabolic profiling revealed distinct subgroups marked by different activated metabolic programs. Patients with predominant activation of glycine/serine metabolism showed favorable grading and a low proliferation index. The second metabolic subgroups were marked by upregulated choline and tryptophan metabolism; it mainly contained high-grade meningiomas but also some lower-grade tumours. The current study is limited by the relatively low number of enrolled patients and the short follow-up time. However, although WHO grade II and III meningiomas are rare, we were able to include 15 patients in this study. A re-evaluation with a longer follow-up should be carried out to confirm these results.

## Conclusion

In summary, our results suggest that tumour malignancy is reflected by metabolic alterations that can support histological classifications in order to predict the clinical outcome of patients with meningiomas. In our study, we investigated choline as a metabolic marker to predict early recurrence and reduced PFS, which is a well-detectable metabolite in in-vivo MR spectroscopy. Further studies are needed to validate this potential correlation in a larger cohort, which would provide a clinical implication for MR spectroscopy in meningioma.

## Supplementary Information

**Additional file 1: Supplementary Figure 1.** A) Heatmap of correlation of module eigengene of module GI and clinical features. B) Metabolic set enrichment analysis (MSEA) of module GI and KEGG pathways. C) Network of metabolites of module GI, nodes and edges were defined by correlation coefficients between all metabolites of module GI. D) A volcano plot reveals differential metabolic intensities between WHO grade I and III meningioma.

**Additional file 2: Supplementary Figure 2.** A) Heatmap containing correlations of module eigengene (module GII) and clinical features. B) Network of metabolites of module GII, nodes and edges were defined by correlation coefficients between all metabolites of module GII. C) Metabolic set enrichment analysis (MSEA) of module GII and KEGG pathways. D) Correlation of module eigengene (module GIII) and clinical features. E) Network analysis of module GIII, nodes and edges were defined by correlation coefficients between all metabolites. F) Metabolic set enrichment analysis (MSEA) of module GIII and KEGG pathways.

**Additional file 3: Supplementary Figure 3.** A-B) Scatter plot of different metabolic intensities between WHO grade I and II (A) and II and III (B), respectively. Colors indicate the corrected *p*-values, cyane points with FDR *p* < 0.05.

## Data Availability

Further information and requests for resources, raw data, and reagents should be directed to and will be fulfilled by DH Heiland (dieter.henrik.heiland@uniklinik-freiburg.de). A full table of all materials is given in the supplementary information.

## Abbreviations

NMR: Nuclear magnetic resonance
WHO: World Health Organization
IGF1R: Insulin-like growth factor 1 receptor
PFK: Phosphofructokinase
LDH: Lactate dehydrogenase
H-NMR: Hydrogen Nuclear Magnetic Resonance
ppm: Parts per million
pQ: Pseudo-counted quantile
WCNA: Weighted correlation network analysis
kME: Intramodular connectivity
MSEA: Metabolite set enrichment analysis
HR-MAS: High resolution magic angle spinning
ROS: Reactive oxygen species
5-HT: 5-Hydroxytryptamine
KYN: Kynurenine
PFS: Progression-free survival
