# Supplementary figures and images for: Metabolic alterations in meningioma reflect the clinical course
Source: BMC Cancer. 2021 Mar 1;21:211. doi: 10.1186/s12885-021-07887-5 (PMC7923818; doi:10.1186/s12885-021-07887-5)

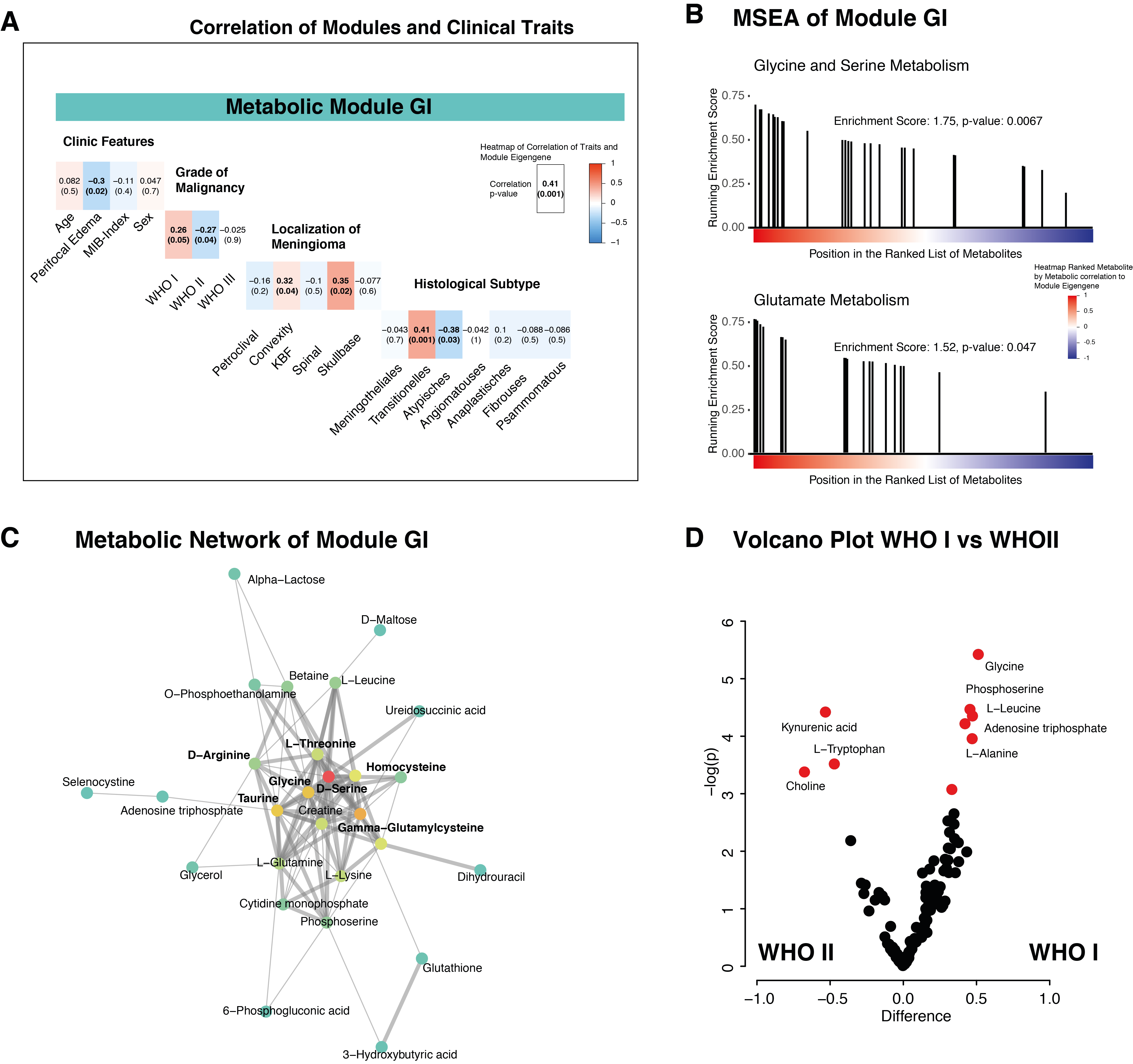

Supplement: Supplementary file 1 — Additional file 1: Supplementary Figure 1. A) Heatmap of correlation of module eigengene of module GI and clinical features. B) Metabolic set enrichment analysis (MSEA) of module GI and KEGG pathways. C) Network of metabolites of module GI, nodes and edges were defined by correlation coefficients between all metabolites of module GI. D) A volcano plot reveals differential metabolic intensities between WHO grade I and III meningioma. [file 12885_2021_7887_MOESM1_ESM.png]

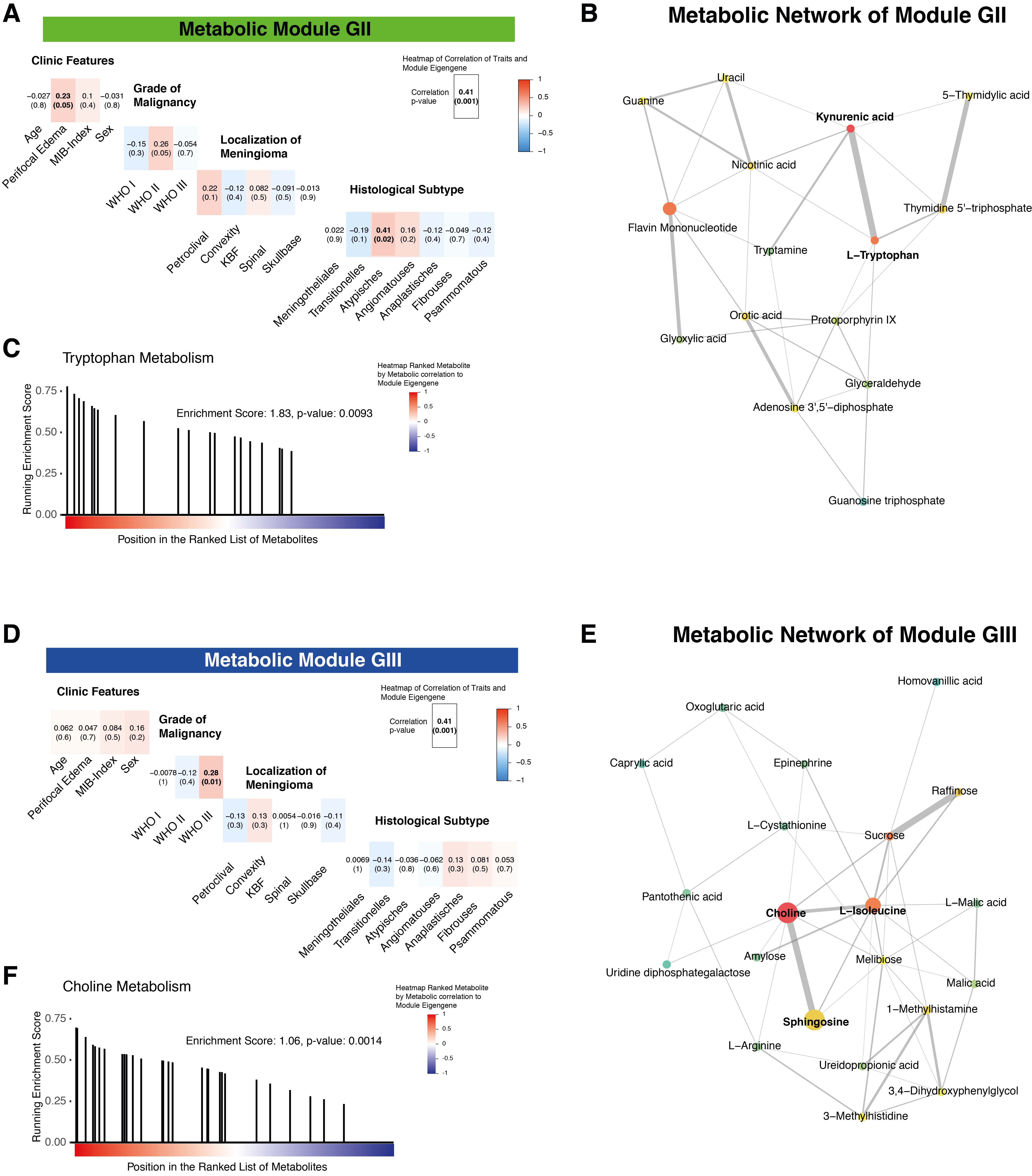

Supplement: Supplementary file 2 — Additional file 2: Supplementary Figure 2. A) Heatmap containing correlations of module eigengene (module GII) and clinical features. B) Network of metabolites of module GII, nodes and edges were defined by correlation coefficients between all metabolites of module GII. C) Metabolic set enrichment analysis (MSEA) of module GII and KEGG pathways. D) Correlation of module eigengene (module GIII) and clinical features. E) Network analysis of module GIII, nodes and edges were defined by correlation coefficients between all metabolites. F) Metabolic set enrichment analysis (MSEA) of module GIII and KEGG pathways. [file 12885_2021_7887_MOESM2_ESM.png]

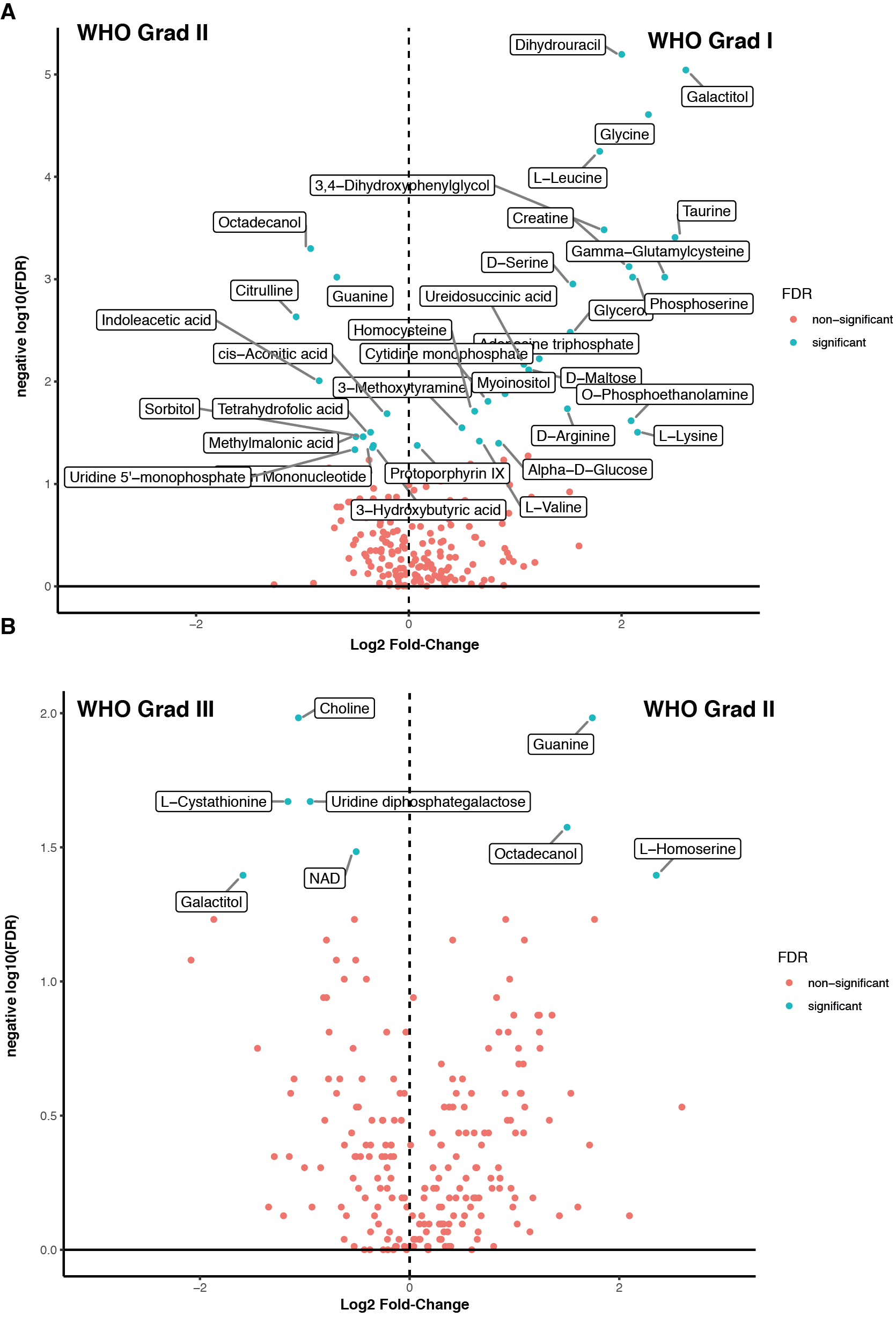

Supplement: Supplementary file 3 — Additional file 3: Supplementary Figure 3. A-B) Scatter plot of different metabolic intensities between WHO grade I and II (A) and II and III (B), respectively. Colors indicate the corrected p-values, cyane points with FDR p < 0.05. [file 12885_2021_7887_MOESM3_ESM.png]
